# The Role of Cardiovascular Magnetic Resonance in Patients with Mitral Regurgitation

**DOI:** 10.3390/jcdd9110399

**Published:** 2022-11-18

**Authors:** Anna Giulia Pavon, Marco Guglielmo, Pierpaolo Mattia Mennilli, Mariana B. L. Falcão, Luca Bergamaschi, David Filip Costantin, Michele Vivaldo, Laura Anna Leo, Susanne Schlossbauer, Christopher W. Roy, Matthias Stuber, Giovanni Pedrazzini, Francesco Faletra

**Affiliations:** 1 Division of Cardiology, Cardiocentro Ticino Institute, Ente Ospedaliero Cantonale, Via Tesserete, 48, 6900 Lugano, Switzerland; 2 Department of Cardiology, Division of Heart and Lungs, University Medical Center Utrecht, Utrecht University, 3584 CX Utrecht, The Netherlands; 3 Department of Radiodiagnostic and Interventional Radiology, University Hospital of Lausanne and University of Lausanne, 1012 Lausanne, Switzerland; 4 Unit of Cardiology, IRCCS Policlinico St. Orsola-Malpighi, Department of Experimental, Diagnostic and Specialty Medicine-DIMES, University of Bologna, 40138 Bologna, Italy; 5 Center for Biomedical Imaging (CIBM), 1012 Lausanne, Switzerland

**Keywords:** mitral valve, cardiovascular magnetic resonance

## Abstract

The 2019 Global Burden of Disease (GBD) study estimated that there were approximately 24.2 million people affected worldwide by degenerative mitral regurgitation (MR), resulting in 34,200 deaths. After aortic stenosis, MR is the most prevalent VHD in Europe and the second-most common VHD to pose indications for surgery in western countries. Current ESC and AHA/ACC guidelines for the management of VHD emphasize the importance of an integrative approach for the assessment of MR severity, which is of paramount importance in dictating the timing for surgery. Transthoracic echocardiography (TTE) and transesophageal echocardiography (TEE) are the first-line imaging modalities; however, despite the technological advancement, sometimes, the final diagnosis on the degree of the disease may still be challenging. In the last 20 years, CMR has emerged as a robust technique in the assessment of patients with cardiac disease, and, recently, its role is gaining more and more importance in the field of VHD. In fact, CMR is the gold standard in the assessment of cardiac volumes, and it is possible to accurately evaluate the regurgitant volume. The purpose of this review is to outline the current state-of-the-art management of MR by using Cardiac Magnetic Resonance (CMR).

## 1. How to “MRI” a Mitral Valve?

**Figure 1:**
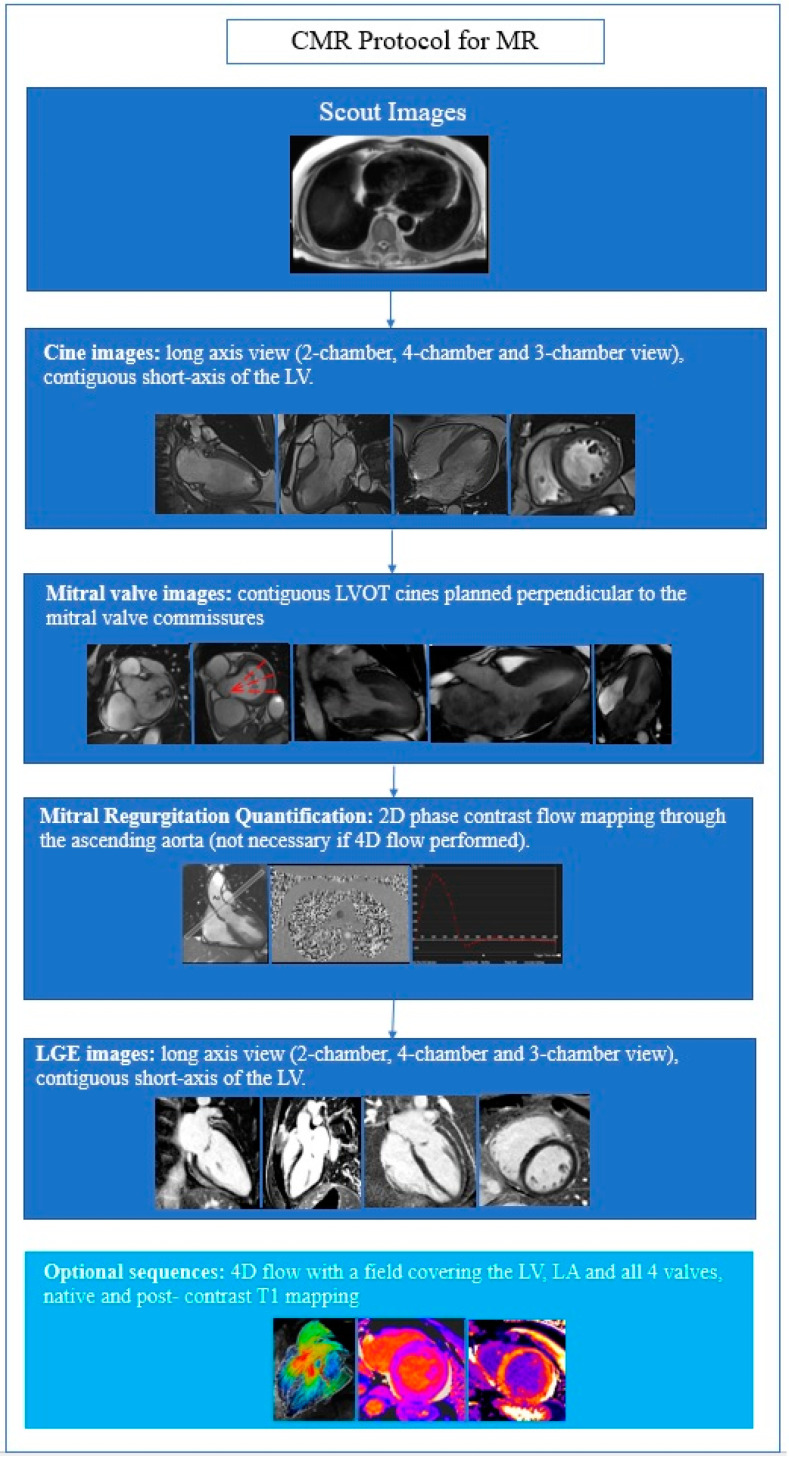
CMR protocol to image a mitral valve. LA: Left atrium; LV: left ventricle; LVOT: left ventricle outflow tract.

The first-line examination for MR remains transthoracic echocardiography (TTE); however, CMR can be of help in many clinical settings [1,2,3]. The aim of a CMR examination focused on MV includes the assessment of MV anatomy and function to quantify the regurgitation and its hemodynamic repercussion on cardiac chambers. Finally, the scanning protocol includes myocardial tissue characterization in order to identify focal and diffuse myocardial fibrosis [4] (Figure 1).

### 1.1. Mitral Valve Apparatus Assessment

**Figure 2:**
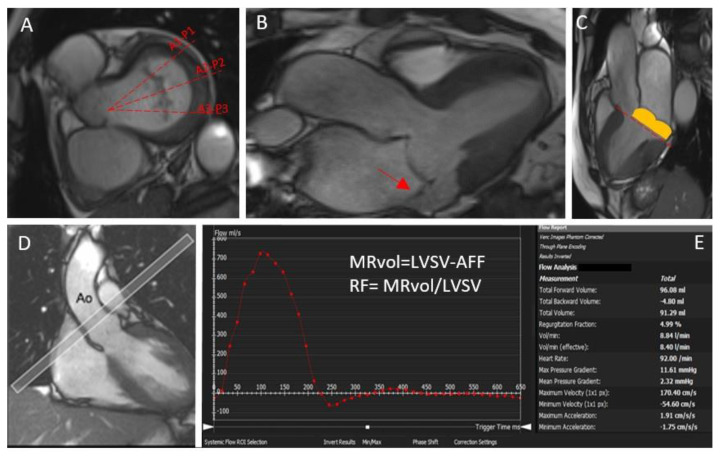
A short axis view of the mitral valve with scallops. Contiguous, long-axis, left ventricular outflow tract SSFP images should be acquired to visualize and assess all the mitral valve cusps following the red lines (**A**). A three-chamber view of a patient with bileaflet mitral valve prolapse in systole (**B**) showing the regurgitant jet (red arrow). A three-chamber view showing the volume of the prolapse that is typically not considered when calculating the ejection fraction ((**C**), orange box). The mitral annular plane is highlighted in red lines. The slice position for the phase-contrast velocity mapping sequence (**D**). (**E**) is showing a flow curve (mL/s) through the ascending aorta to calculate the aortic forward flow. MRvol = mitral regurgitant volume; AFF = aortic forward flow; LVSV = left ventricle stroke volume.

MRI can be used to assess leaflets’ anatomy and movement. The steady-state free precession (SSFP) pulse sequence is commonly used in this setting since it provides a good assessment of valve morphology and function [5]. Image acquisition is ECG-gated, and each slice is obtained during a single breath-hold of 5–8 s [5]. A visual assessment should be made of all four components of the mitral valve: the anterior leaflets, the posterior leaflets, the annulus, and the sub-valvular apparatus enclouding the papillary muscles [5]. Contiguous, long-axis, left ventricular outflow tract SSFP images should be acquired to visualize and assess all the mitral valve cusps (A1-P1, A2-P2, and A3-P3) [4] (Figure 2A). In this way, it is possible to detect the mechanism of the MR, detecting the presence of prolapsing leaflets or a functional MR. Nevertheless, it must be pointed out that small, thin structures (i.e., fibroelastomas or vegetations) may not be correctly visualized due to spatial resolution.

### 1.2. Ventricular Volume and Function

Correctly imaging the left ventricular (LV) volume and function is of paramount importance in assessing the hemodynamic repercussion of the valvulopathy and avoiding the progression through cardiac dysfunction. Finally, therefore, it is essential to decide on the timing for surgical intervention [1,2] (Figure 2B). Notably, CMR is the gold-standard imaging technique for evaluating LV volume, mass, and function. LV imaging should be assessed according to SCMR guidelines [5]. Ventricular volumes are calculated from a short-axis stack of 6–8 mm thick slices with an interslice gap of 4 mm [5]. It must be taken into account that, in patients with prominent mitral valve prolapse (MVP), the echocardiographic quantification made by Simpson’s method may underestimate the LV end-systolic volume (LVESV), as it only considers the volume located between the apex and the mitral annulus and neglects the ventricular volume that is displaced into the left atrium but is contained within the prolapsed mitral leaflets at the end systole [6]. This may lead to an underestimation of LVESV, with a consequent overestimation of mitral regurgitation [7]. The recent series published by Vincenti G et al. [8] highlighted how, for patients with severe bileaflet prolapse, the correction of the LVSV considering the prolapse volume leads to a lower LV ejection fraction, and it also modifies the assessment of MR severity by one grade in a large portion of patients (Figure 2C). However, whether this has prognostic value still needs further validation.

### 1.3. Tissue Characterization

CMR is the only cardiac imaging technique allowing for the evaluation of myocardial composition. The cornerstone of this aspect is Late Gadolinium contrast-enhanced (LGE) imaging, which is typically obtained 10–15 min after contrast media injection [5]. Since gadolinium contrast media are able to reduce the T1 relaxation time and have a different wash-out in the normal myocardium compared to the pathological myocardial area, it highlights the presence of a damaged myocardium. The latter has a brighter signal, in contrast with the dark healthy myocardium [9]. Another technique that allows for a more quantitative approach to tissue characterization is T1 mapping. Higher native T1 relaxation times may be caused by edema, fibrosis, or protein accumulation. Finally, post-contrast T1 mapping, together with the hematocrit levels, is essential for extracellular volume (ECV) quantification [5].

### 1.4. Mitral Regurgitation Quantification

The quantification of the MR is a fundamental part of the CMR examination [1,4]. Several qualitative and quantitative methods are available. Starting from qualitative assessment, the MR jet can be visualized using both cine and 2D phase-contrast CMR. This is a visual approach which relies on spin dephasing in cine images. However, it must be pointed out that, given its susceptibility and the important intra-observer and inter-observer variability, this method should be used only to assess the presence of MR, while any estimation of the degree of severity should be performed only quantitatively [4].

#### 1.4.1. 2D Phase Contrast (PC) Velocity Mapping

2D PC velocity mapping is the cornerstone of flow imaging and represents the standard approach to MR quantification [9]. The preferred sequence parameters of 2D PC imaging include a though-plane image placed at the sino-tubular junction in the end-diastole to quantify forward flow (Figure 2D,E). This plane should be perpendicular to the vessel. The baseline velocity encoding for aortic flow is 2.0–2.5 m/s, and the temporal resolution is 25–45 ms. The standard method to calculate the MR volume lies in 2D PC velocity mapping and accounts for the difference between the LV stroke volume calculated using the planimetry of SSFP and the forward volume obtained by 2D PC images [5]. In this way, it is possible to calculate the regurgitant volume and the regurgitant fraction. The mitral regurgitant volume (MRvol) is expressed as the difference between the left ventricular stroke volume (LVSV) and the aortic forward flow (AFF), and the regurgitant fraction (RF) is the MRvol divided by the LVSV, expressed as a percentage. According to both American and European recommendations, an RF > 50% or an MRvol > 60 mL identifies patients with severe MR. As can be imagined, the crucial advantages of this method account for the easy measurement, the lack of a need for geometric assumption, the lack of a contrast agent application, and the short investigation time [10]. However, it must be noted that the accuracy of the PC measurement can be reduced in cases of irregular rhythm or patient motion. Additionally, this standard 2D-based approach does not allow for visualizing or quantifying the 3D structure of the regurgitant jets, as, for instance, echocardiography does.

#### 1.4.2. Quantitative Assessment of MR with CMR

The MRvol and MR RF can be measured according to different methods using a combination of 2D phase contrast velocity mapping and cine images.

1. The standard approach implies the quantification of MRvol and MR RF considering the difference between the LV stroke volume calculated using the planimetry of SSFP images and the aortic (systolic) forward volume obtained by phase-contrast images (AoPC).
2. If no other valve regurgitation or haemodynamically significant shunt are present, the MRvol can be derived by the difference between the LV stroke volume and the RV stroke volume calculated using the planimetry of SSFP images. It must be noted that, given the relatively lower precision with which the RV stroke volume is quantified compared with the LV stroke volume, intra- and inter-observed variability is lower compared with other methods [11].
3. The difference between the mitral inflow stroke volume and the AoPC. If this method is suitable for patients with multiple valve regurgitations, the fact that 2D phase-contrast CMR requires static imaging planes and cannot adapt to valve motion may result in some inaccuracy [12].
4. Finally, if 4D-flow is available, a direct quantification of the MR flow with retrospective mitral valve tracking can also be performed. MR jets are quantified by defining a systolic reformatted plane perpendicular to the single jet or individually for multiple jets. Otherwise, a reconstructed aortic plane using the retrospective valve-tracking method can be used to quantify AoPC. This measurement can then be used to quantify the MR volume or fraction using the standard LVSV–AoPC method.

#### 1.4.3. 4D Flow

4D flow velocity-encoded CMR imaging is an emerging technique that involves phase-contrast acquisition with flow encoding in all three spatial directions and to the dimension of time. New advances in 4D flow MRI [13] are growing, enabling a more in-depth quantification of MR. Research on this modality has been growing over the past years, and it has enabled not only the quantification of aortic forward flow but also the valve tracking, indirect regurgitant flow tracking, regurgitant jet quantification, and computation of mitral forward flow [14,15]. In a study by Fidock et al. in 2021 [16], 35 patients with different levels of MR severity were recruited for CMR, including the acquisition of 2D cines and 4D flow, and four different methods for MR quantification were compared. Patients were distributed into three groups: having Primary MR, having secondary MR, and having had mitral valve replacement (MPV). The most reproducible technique was reported to be regurgitation assessment based on the correlation between the mitral inflow and aortic inflow, both measured directly from 4D flow MRI data. In 2020, Blanken et al. [17] compared valve tracking and flow tracking semiautomatic quantification techniques to investigate the reproducibility of each measurement in 34 MR patients with different MR severity diagnoses, and they concluded that flow tracking of the regurgitation jets provided more accurate quantification of MR in terms of agreement with the indirect reference measurement, particularly for severe MR cases [17]. Notably, with the advances in accelerated 4D flow MRI, the scan time could possibly be reduced whilst maintaining the accuracy in results. For instance, in 2022, Blanken et al. [18] introduced a new sampling pattern for acquiring a faster 4D flow CMR for valvular quantification, enabling whole heart acquisitions in a short scan time (<10 min) with reliable MR quantification. Additionally, the introduction of the so-called free-running techniques could become a valuable aid in simplifying the scanning workflow, as they acquire imaging data without interruptions over a fixed period and without the need for complex slice positions or navigator placement [18,19]. Nevertheless, a validation of these techniques for MR quantification is still lacking.

However, in contrast to the previous decade, several options of analysis tools for 4D flow are available, and, to date, a standardized analysis process that allows for a uniform workflow and will generate reproducible and comparable quantification is lacking [4]. 

Clearly, in clinical settings, 4D flow will not replace echocardiography, but since it can provide a reliable quantification of MR when compared with 3D transesophageal echocardiography and has the direct advantages of a non-invasive technique that does not require sedation, it should be considered as a compliment technique. Additionally, it can be used in patients with comorbidities that prevent the use of transesophageal echocardiography [12]. 

## 2. Echocardiography and Cardiovascular Magnetic Resonance: Friends or Foes?

In patients with MR, agreement between CMR and the echocardiographic guidelines algorithm was suboptimal. Several studies have shown that echocardiography has limitations in distinguishing non-severe MR from severe MR [20]. Frequently, there could be discordance among the echocardiographic parameters of MR severity, and there was no guideline-defined hierarchy for weighing individual parameters [20,21,22]. Recently, S. Uretsky et al. found that, among patients identified as having severe MR by the American Society of Echocardiography algorithm, less than half had severe MR by concomitant CMR evaluations [23]. This finding is consistent with prior studies that have shown a significant discordance between CMR and echocardiography, with absolute agreement values ranging from 36% to 63% [24,25]. 

The ‘internal coherence’ of the various measures is of paramount importance in the assessment of thr LV volume and quantitative parameters of MR grading. In fact, in routine echocardiographic practice, the occurrence of these discrepancies between effective stroke and regurgitant volume are not infrequent. In this setting, the CMR evaluation could improve the internal coherence in the MR assessment thanks to its high accuracy and reproducibility through a combination of LV volumetric measurements and aortic flow quantification with phase-contrast velocity mapping or with newer tools such as 4D flow cardiac MRI [3]. 

Finally, regurgitant volume by CMR seems to be the most reliable method for identifying patients with severe MV, as it is an independent predictor of LV reverse remodelling after mitral valve correction compared to other echocardiographic parameters [23].

The main remaining pitfalls in the assessment of MR by CMR are the fact that current severity thresholds are derived from echocardiographic data and the lack of standard cut-off values. However, recent studies suggest that CMR-specific thresholds may be more appropriate and more closely related to outcome [26], and the increased reproducibility and accuracy of LV and MR quantification may pave the way for the routine use of CMR in the assessment of mitral valve disease [27].

## 3. What Is the Role of CMR in Primary Mitral Regurgitation?

In both European and AHA/ACC guidelines for the management of MR, CMR is considered only in cases of an inconclusive echocardiography [1,2]. Moreover, no specific indications regarding additional information that can be provided by CMR according to the aetiology of MR are provided.

It must be noticed that, to date, if CMR is superior in assessing cardiac volume and function compared to echocardiography, a gold standard in the quantification of MR severity is still lacking [1]. Surely, echocardiography remains the first-line examination to evaluate primary MR; however the quantification of MR may be challenging, since it strongly relies on an acoustic window and geometric assumptions [1]. To date, only a few studies have tried to compare the degree of severity of primary MR detected in TTE with CMR, showing no conclusive results [28,29]. As mentioned, Uretsky et al. [20] recently demonstrated that there is limited concordance between the echocardiographic parameters of MR severity, and the discordance was worse with more severe MR, highlighting the challenges facing echocardiographers when assessing the severity of MR and emphasizing the need for using an integrated approach that incorporates multiple components. Moreover, the evaluation of MR in CMR has been proven to have a prognostic role. The first larger prospective study in primary MR was published by Myerson et. al. in 2016 [26]. In this landmark paper, patients with moderate or severe primary MR detected in TTE were followed up for a mean time of 2.5 ± 1.9 years. In this cohort of 109 asymptomatic patients, the presence of a regurgitant volume >55 mL was found to be associated with the appearance of symptoms and the need for surgery [26]. Moreover, an MR regurgitant volume > 55 mL was also found to be the best predictor of mortality in a prospective, multicentric study involving 258 asymptomatic patients with moderate or severe primary MR [27]. 

### Arrhythmic Mitral Valve Prolapse

MVP is a common cardiac condition, with an estimated prevalence between 1% and 3% [30]. Most of the patients with this condition have a benign course, but ever since its initial description, mitral valve prolapse has been associated with higher risk of complex ventricular arrhythmia and sudden cardiac death [31]. Arrhythmogenesis in patients with mitral valve prolapse is a complex interplay between different factors which is still not completely deciphered. The fundamental role of cardiac imaging is to highlight the presence of high-risk features, which possibly expose patients to a higher risk of arrhythmic complications [32]. The echocardiographic “high-risk” features include the presence of a bileaflet MVP (defined as the presence of a displacement >2 mm beyond the long-axis annular plane, with >5 mm leaflet thickening [33]) accompanied by morphofunctional abnormalities of the mitral annulus such as mitral annular disjunction (MAD) and systolic curling [31]. Conventionally, MAD is defined as the apparent systolic separation of the mitral leaflet insertion from the ventricular myocardium, typically evaluated in a three-chamber view [34]. However, it must be pointed out that the presence of MAD has a circumferential extension, and, sometimes, its identification with TTE will be challenging [31]. On the contrary, the extent of the longitudinal MAD distance located in the posterolateral wall is easily assessed by CMR. Essayagh et al. compared TTE and CMR for the detection of MAD and found a low sensitivity (65%) but a high specificity (96%) for TTE [35]. A recent study compared TTE, TEE, and CMR and showed only a moderate agreement between TTE and CMR, while a good agreement was found between TEE and CMR [36]. Notably, no specific analysis on the patho-morphomogy of MAD exists so far. Recently, evidence for the fact that MAD can also be present in normal hearts has been described and highlights the fact that the role of MAD in arrhythmogenesis may be in need of further analysis [37,38]. In particular, it is possible to distinguish two scenarios: the first scenario with “pseudo-MAD” (sited on P2 insertion), in which MAD is detected only in the systole and actually does not exist, it being formed from the juxtaposition of the belly of the billowing posterior leaflet on the adjacent left atrial wall, giving the illusion that a disjunction is present, but a normal attachment of the leaflet can be observed in the diastolic phase. A second scenario is a “true-MAD”, in which the disjunction can be seen in both the systole and diastole, and it is linked to an abnormal attachment of the leaflet in the atrial wall. However, how these two entities may correlate with arrhythmias still needs further study [39].

**Figure 3:**
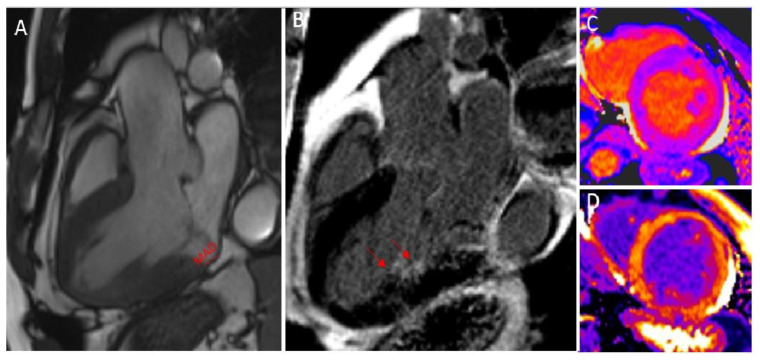
CMR in a patient with MVP. MAD can be clearly diagnosed in an SSFP three-chamber view in the systole (red lines) (**A**). The presence of fibrosis on the tip of papillary muscles (red arrow) is highlighted in LGE (**B**); finally, higher levels of interstitial fibrosis can be identified by higher levels of native T1 mapping (**C**) and ECV after post-contrast T1 mapping (**D**).

The other fundamental feature defining the “arrhythmic mitral valve prolapse” is the presence of fibrosis, traditionally detected in LGE sequences at the level of the inferior wall, the infero-lateral wall, and papillary muscles in the landmark paper of Basso C et al. [40], confirmed in several following studies [41,42] (Figure 3). The fact that MVP influences the ventricular remodeling has recently been confirmed by Costant Dit Beaufils et al. [43], showing that replacement myocardial fibrosis was present in 28% of patients with various degrees of MVP and that it was especially located in the basal inferolateral wall or papillary muscle. The myocardial fibrosis prevalence was 13% in trace-mild MR, 28% in moderate MR, and 37% in severe MR, and it was associated with specific features of the mitral valve apparatus, a more dilated LV, and more frequent ventricular arrhythmias (45% vs. 26%, *p* < 0.0001) [43]. The study highlights how the presence of fibrosis, associated with mitral valve apparatus alterations, was finally associated with a more dilated LV, a higher MR degree, and ventricular arrhythmia—all features that are independent risk factors for adverse cardiovascular events. 

The future holds promising scenarios, and, apart from localized areas of fibrosis demonstrated on LGE, the presence of interstitial fibrosis evaluated by native T1 mapping or ECV has also been recently analyzed for better arrhythmic risk stratification [41,44,45]. In this setting, patients with MVP were found to have a higher degree of ventricular remodeling, going beyond what can be detected in LGE sequences, evidenced by the higher native T1 relaxation time or higher ECV. Moreover, an ECV > 33% was found to have the same predictive value regarding out-of-hospital cardiac arrest as LGE [41]. 

## 4. What Is the Role of MRI in Secondary Mitral Regurgitation?

Secondary MR is the result of LV dysfunction that can be due to various diseases, following myocardial infarction most of the time [1]. In this setting, CMR examinations can provide an accurate assessment of LV dysfunction, highlighting the presence of myocardial scar/necrosis and the viability of myocardial segments and providing important clues that are useful in the work-up of dilated cardiomyopathies [4,46]. This is particularly important for patients who are candidates for surgical revascularization, in which a concomitant surgical treatment of the MV is also suggested. In a pilot study published in 2009, it was first highlighted how posterior papillary muscle region scarring severity correlated with decreased segmental wall motion and a poorer mitral regurgitation correction after coronary revascularization and annuloplasty, suggesting that routinely assessing scar burden may help to identify patients for whom annuloplasty alone is insufficient to eliminate mitral regurgitation [47]. Moreover, it must be noticed that the progression of ischemic mitral regurgitation was also found to be independently associated with adverse LV remodeling and infarct size [48]. 

It is well known that ischemic mitral regurgitation is typically associated with poor outcomes [1]. In this context, it can be speculated that knowing the viability of the myocardial wall and the possible improvement of myocardial contraction after revascularization may also lead to an improvement in the degree of the MR. However, to date, no specific large studies have been published regarding how the use of CMR may influence clinical management in this specific clinical setting.

Finally, it must be considered that, in recent years, the percutaneous treatment of MR with the MitraClip^TM^ system has also become more and more available in patients with secondary MR [1]. Given the fact that the procedure is relatively new, no studies regarding the use of CMR in the work-up of possible candidates have been published so far [49]. However, in a small series of patients, CMR has been shown to perform very well in the quantitation of MR after MitraClip^TM^ insertion, with an excellent reproducibility compared to echocardiographic methods. Moreover, the evidence that MitraClip^TM^ implantation may be associated with LV reverse remodeling may open the question on the role of CMR tissue characterization in the screening of possible candidates that can benefit from the procedures 

## 5. Future Steps

The role of CMR in the setting of MR evaluation is rapidly expanding as a complement technique to echocardiography to improve the patient’s evaluation. Surely, the novelty of 4D flow, allowing for the quantification of flow and regurgitation in many novel ways, will be of help in the future. These methods could include particle tracing component analysis, intra-cavity energetics, retrospective valve tracking to quantify forward and regurgitant jets, 4D flow-derived parameters, semi-quantitative streamline visualization, and intra-cavity hemodynamic forces [50,51]. However, the possible role of these methods and their prognostic relevance still need to be validated.

Moreover, if, on the one hand, it is well known that CMR is the gold reference standard for evaluating volume and function with the possibility for MR quantification that conveys information, and it is possible to quantify MR with a clinical outcome benefit over echocardiography, it must be noted that it still remains unclear which method for MR quantification is more reliable in clinical practice. Finally, despite the fact that the 4D flow technique has also shown promising results, future studies are needed to prospectively evaluate the clinical outcome benefit of using 4D flow CMR for MR quantification in challenging situations.

## 6. Conclusions

CMR has become a robust and reliable imaging modality, not only for the assessment of ventricle structure and function but also for the quantification of MR. Growing evidence shows that CMR could be an accurate complementary method to echocardiography for grading regurgitation severity. Using comprehensive techniques, CMR allows for an accurate measurement of valvular regurgitant volume (Rvol) and regurgitant fraction (RF), independent of jet morphology and direction. Furthermore, due to its unique ability to assess focal and diffuse LV fibrosis, CMR provides potential information in the clinical evaluation of MR for planning and deciding on the timing and indication of a specific therapy or intervention. Accordingly, taking into consideration the potential accurate uses of CMR in the evaluation of MR, this imaging modality could be useful in the assessment of the unclear severity of valvular regurgitation measured by echocardiography.

## Data Availability

Not applicable.
